# Successful Treatment of Refractory Mycobacterium avium Complex Pulmonary Disease With Sitafloxacin After Failed Amikacin Liposome Inhalation Suspension Therapy

**DOI:** 10.7759/cureus.77645

**Published:** 2025-01-18

**Authors:** Maiya Chen, Toyoshi Yanagihara, Natsumi Kushima, Takato Ikeda, Yuki Shundo, Naoki Hamada, Masaki Fujita

**Affiliations:** 1 Department of Respiratory Medicine, Fukuoka University Hospital, Fukuoka, JPN

**Keywords:** amikacin liposome inhalation suspension (alis) resistance, fluoroquinolone, mycobacterium avium complex (mac), refractory to standard treatment, sitafloxacin

## Abstract

*Mycobacterium avium* complex (MAC) pulmonary disease presents significant therapeutic challenges in respiratory medicine. While amikacin liposome inhalation suspension (ALIS) has emerged as a promising option for treatment-refractory cases, some patients continue to show persistence of infection. We report a case of a 64-year-old male with MAC pulmonary disease who demonstrated a remarkable clinical response to sitafloxacin (STFX) after failing both guideline-based therapy and subsequent ALIS treatment. Although ALIS initially achieved temporary culture conversion, the patient experienced disease recurrence. Following the initiation of STFX, the patient showed significant clinical improvement with resolution of hemoptysis within three months, achieving sustained culture conversion, which has persisted for over two years. This case highlights the potential role of STFX as an effective therapeutic option in the management of refractory MAC pulmonary disease, particularly in cases where guideline-based treatments, including ALIS, have failed.

## Introduction

*Mycobacterium avium* complex (MAC) pulmonary disease is a significant clinical practice therapeutic challenge. The current standard of care consists of a triple-drug combination therapy, centered on macrolides, for patients with macrolide-susceptible MAC pulmonary disease [1]. Despite this established approach, treatment success rates remain suboptimal, with significant numbers of patients experiencing persistent or recurrent infection. The recent approval of amikacin liposome inhalation suspension (ALIS) has provided a new therapeutic option for refractory cases [2]. However, clinical experience has shown that some patients continue to demonstrate disease progression even after ALIS therapy, highlighting the urgent need for alternative treatment strategies. Sitafloxacin (STFX), a fourth-generation fluoroquinolone with broad activity against a wide range of Gram-negative and Gram-positive bacteria, was developed and launched in Japan in 2008. Through its potent inhibitory activity against both DNA gyrase and topoisomerase IV enzymes, it demonstrates superior antimicrobial activity compared to conventional quinolones [3]. STFX has also shown promising antimycobacterial activity in vitro [4], but clinical evidence for its efficacy in refractory MAC disease remains limited. Here, we present a case of successful treatment with STFX in a patient with MAC pulmonary disease that persisted despite guideline-based therapy and ALIS treatment, along with a comprehensive review of the relevant literature.

## Case presentation

**Figure 1:**
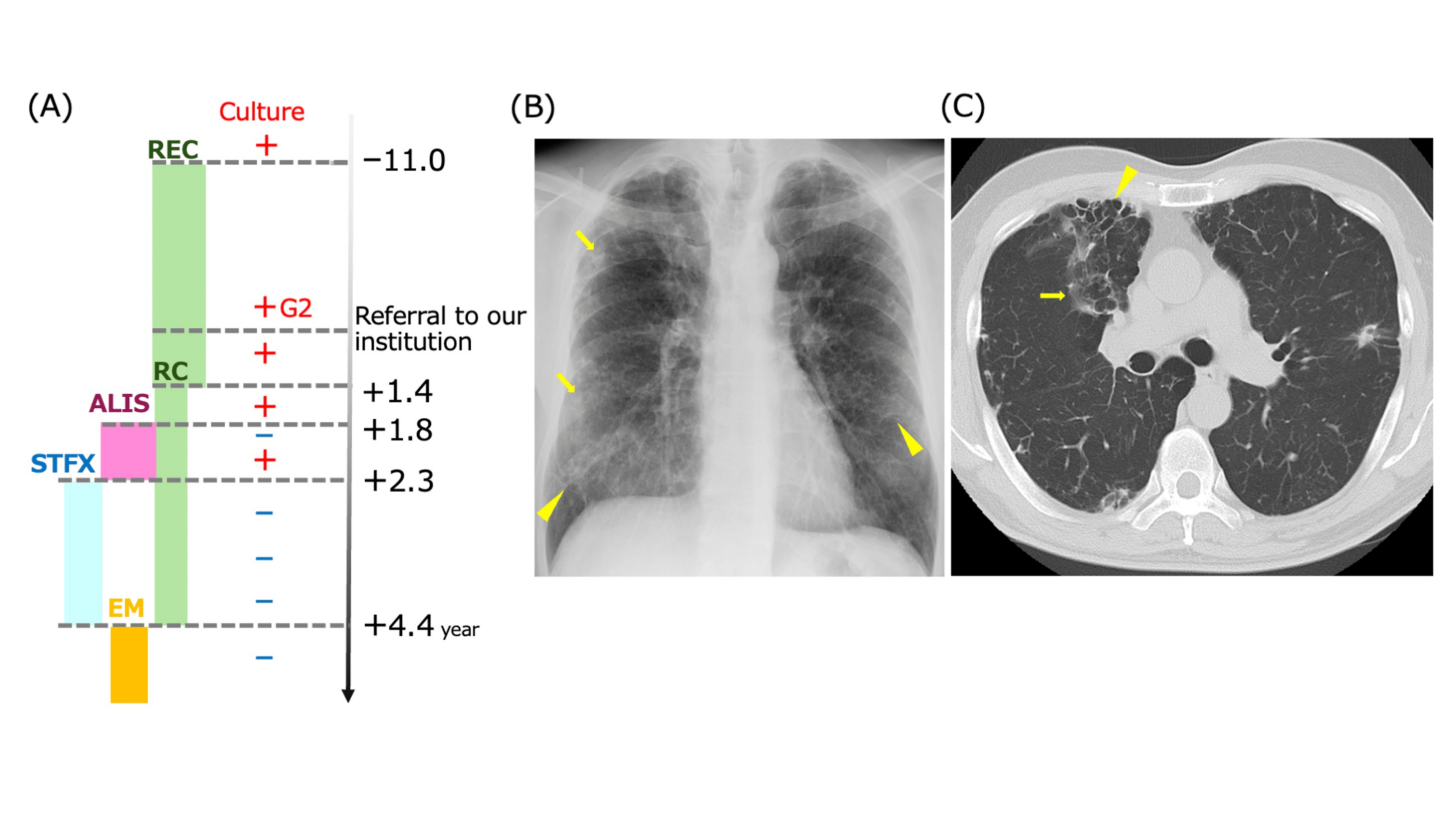
Clinical course and radiological findings. (A) Timeline of treatment and culture results. The vertical axis shows different treatment regimens: REC (green) represents the initial triple-drug therapy (CAM + EB + RFP), RC (green) shows the period after EB discontinuation, ALIS (pink) indicates amikacin liposome inhalation suspension therapy, STFX (blue) shows sitafloxacin treatment period, and EM (yellow) represents erythromycin maintenance therapy. The right side shows the time course in years from referral. Plus (+) and minus (-) signs indicate positive and negative sputum cultures, respectively. G2 represents Gaffky scale 2. (B) Chest X-ray at the initial presentation showing nodular (arrows) and ground-glass opacities (arrowheads) in both lungs. (C) Chest CT scan at the initial presentation demonstrating bronchiectasis (arrowhead), nodular shadows (arrow), and infiltrates predominantly in the right lung.

A 64-year-old Japanese male resident was referred to our hospital with a persistent cough and occasional bloody sputum. He had been diagnosed with MAC pulmonary disease 11 years prior at another hospital. He had been receiving standard triple therapy consisting of clarithromycin (CAM) 800 mg, ethambutol (EB) 750 mg, and rifampicin (RFP) 450 mg. However, his cough and bloody sputum persisted, alternating between periods of improvement and exacerbation (Figure 1A). His medical history included mild chronic obstructive pulmonary disease (COPD), which was not being treated with inhalation therapy. Notably, he had no other risk factors for pulmonary MAC disease, such as bronchiectasis, interstitial pneumonia, history of tuberculosis, asthma, or solid tumors. Additionally, there were no coexisting conditions that could cause immunodeficiency. He had no history of smoking, alcohol consumption, or allergies. No family history of tuberculosis, allergies, or other significant medical conditions existed.

On examination, his body mass index was 22.5 kg/m^2^. He was afebrile, and other vital signs were within normal limits. Breath sounds were clear bilaterally, with no adventitious sounds. No clubbing was observed. Initial laboratory findings revealed a C-reactive protein level of 0.21 mg/dL and an elevated MAC antibody titer of 1.23 U/mL (reference <0.70) (Table 1). Sputum cultures were positive for *Mycobacterium intracellulare* with a Gaffky scale of 2. Drug susceptibility testing demonstrated sensitivity to CAM (minimum inhibitory concentration [MIC] 0.25) and RFP (MIC 0.25), with intermediate sensitivity to amikacin (MIC 8) and levofloxacin (MIC 2). Chest radiography revealed nodular and ground-glass opacities in both lungs. Chest computed tomography demonstrated bronchiectasis, nodular shadows, and infiltrates predominantly in the right lung (Figures 1B, 1C).

**Table 1:** Laboratory findings. Units are provided in parentheses: n/L, per liter; g/dL, grams per deciliter; mg/dL, milligrams per deciliter; mEq/L, milliequivalents per liter; U/L, units per liter. WBC, white blood cell count; RBC, red blood cell count; Hb, hemoglobin; Plt, platelets; Alb, albumin; CRP, C-reactive protein; AST, aspartate aminotransferase; ALT, alanine aminotransferase; BUN, blood urea nitrogen; Cr, creatinine; Na, sodium; K, potassium

Test	Value	Reference range
WBC (/μL)	6,600	3,300-8,600
RBC (10^4/μL)	577	435-555
Hb (g/dL)	17.3	13.7-16.8
Plt (10^3/μL)	242	158-348
Alb(g/dL)	4.5	6.6-8.1
CRP (mg/dL)	0.21	0.4-1.5
AST (U/L)	30	13-30
ALT (U/L)	35	10-42
BUN (mg/dL)	18	8-20
Cr (mg/dL)	0.71	0.65-1.07
Na (mEq/L)	141	138-145
K (mEq/L)	4.3	3.6-4.8

The patient continued on triple-drug therapy until 1.4 years after the initial presentation when EB was discontinued due to diplopia. At 1.8 years, ALIS was initiated due to persistent infection. However, ALIS was discontinued after six months (2.3 years from the initial presentation) due to failure to achieve culture conversion and persistent bloody sputum. Treatment was then modified to include STFX 100 mg, along with CAM and RFP. Following this change, the patient experienced a resolution of bloody sputum and achieved culture conversion within three months. The combination therapy of STFX, CAM, and RFP was maintained until 4.4 years after the initial presentation, after which the patient transitioned to maintenance therapy with erythromycin monotherapy. Cultural negativity was maintained throughout the follow-up period.

## Discussion

The management of refractory MAC pulmonary disease remains challenging, particularly in cases that fail to respond to both conventional therapy and newer treatment options. The current treatment landscape for refractory Mycobacterium avium complex (MAC) pulmonary disease, as outlined in the 2020 revised guidelines by the American Thoracic Society (ATS), European Respiratory Society (ERS), European Society of Clinical Microbiology and Infectious Diseases (ESCMID), and Infectious Diseases Society of America (IDSA), recommends the addition of aminoglycosides (either ALIS, intravenous amikacin, or intramuscular streptomycin) for cases resistant to standard therapy [1]. However, some patients fail to respond even to these recommended salvage therapies. The dramatic and sustained response to STFX in our patient, who had failed both conventional therapy and ALIS, suggests that STFX may represent a valuable alternative for highly refractory cases.

The efficacy of STFX demonstrated in our case is supported by both preclinical and clinical evidence in the literature. Preclinical studies have established the superior antimicrobial activity of STFX against *M. avium* compared to other fluoroquinolones. Sano et al. demonstrated this superiority through both in vitro studies and in vivo experiments using infected mouse models in 2011 [5]. Their research revealed that STFX not only exhibited potent activity as a single agent but also showed significant synergistic effects when combined with CAM and EB, particularly in bacterial elimination from both pulmonary and splenic tissues. Notably, in a comparative analysis of new quinolones (including ciprofloxacin, gatifloxacin, levofloxacin, moxifloxacin, sparfloxacin, and tosufloxacin), STFX demonstrated the lowest MIC of 1 μg/mL against MAC, based on testing of 16 strains (eight *M. avium* and eight *M. intracellulare* isolates) [6]. This superior antimicrobial activity provides a strong pharmacological rationale for its clinical application in refractory MAC disease.

Clinical evidence supporting STFX's efficacy has also emerged from multiple studies. Fujita et al. retrospectively analyzed 18 patients with recurrent or refractory pulmonary MAC disease treated with STFX-containing regimens. Their findings were promising: 55.6% of patients demonstrated radiological improvement, and 44.4% achieved sputum culture conversion within six months [7]. A subsequent larger study by Asakura et al. provided additional evidence for STFX's long-term efficacy and safety. Among patients receiving STFX-containing regimens, 26% showed symptomatic improvement and 19% demonstrated radiological response at 12 months [8]. This study also established STFX's favorable safety profile during extended use. Our case aligns with and extends these findings, demonstrating particularly rapid clinical improvement with the resolution of hemoptysis and achievement of culture conversion within just three months of STFX initiation, with sustained response over two years. Regarding the starting dose of STFX, different approaches are supported by the literature. Fujita et al. revealed that 94% (17/18) of patients started at 100 mg/day, with two patients later requiring dose reduction to 50 mg/day [7]. In contrast, Asakura et al. reported initiating STFX at 200 mg/day in 90% of patients [8]. The patient in this case report showed therapeutic response at 100 mg/day, eliminating the need for dose escalation. If the response had been inadequate, increasing to 200 mg/day would have been our next step.

Given the patient's relatively young age (60s) and focal bronchiectasis on CT images, surgical resection of the affected lung represents a viable therapeutic option for this ALIS-refractory case, as recommended by the current guideline [1]. While we initially opted for STFX treatment, surgical intervention remains an important consideration if medical management proves insufficient to control the disease.

Several important limitations need to be considered. First, STFX is not widely recognized as a treatment option in global guidelines, as it is currently only available in limited regions such as Japan and Thailand. This geographical restriction has led to a paucity of international clinical data. Second, we did not measure the MIC for STFX in the case, making it difficult to evaluate antimicrobial efficacy and establish optimal dosing strategies. Finally, long-term fluoroquinolone use raises concerns about the development of drug resistance, not only in MAC but also in other microorganisms, particularly in patients with concurrent infections such as *Pseudomonas aeruginosa*. These limitations highlight the need for further research, including larger prospective studies with systematic MIC measurement and long-term follow-up. Until then, STFX should be considered as a potential salvage therapy option, particularly in regions where it is available and in cases where standard treatments have failed.

## Conclusions

STFX may serve as a promising salvage therapy option for refractory MAC pulmonary disease, particularly in cases that have failed both conventional therapy and ALIS treatment, as demonstrated by the rapid clinical improvement and sustained culture conversion in our case. However, significant limitations, including its limited geographical availability, lack of MIC measurements, and concerns about long-term fluoroquinolone resistance, necessitate careful consideration of its use. Larger prospective studies with systematic evaluation of antimicrobial susceptibility are needed to establish the optimal role of STFX in the treatment algorithm for refractory MAC pulmonary disease.
